# Usefulness of albumin-to-D-dimer ratio in predicting the long-term outcome after hospital discharge in patients with ST-elevation myocardial infarction: a retrospective cohort study

**DOI:** 10.3389/fcvm.2026.1629253

**Published:** 2026-02-27

**Authors:** Guangze Xiang, Huanyi Zhou, Dongjie Liang, Jia He, Peiren Shan

**Affiliations:** Department of Cardiology, Heart Center, First Affiliated Hospital of Wenzhou Medical University, Wenzhou, China

**Keywords:** albumin, albumin-to-D-dimer ratio, all-cause mortality, D-dimer, new-onset stroke, ST-elevation myocardial infarction

## Abstract

**Objective:**

The Albumin-to-D-dimer ratio (ADR), a novel systemic inflammatory marker, has been linked to adverse outcomes in patients with cardiovascular disease. However, limited research has explored its prognostic value in ST-elevation myocardial infarction (STEMI) survivors following hospital discharge. This study aimed to evaluate the prognostic significance of ADR in hospital-discharged STEMI patients.

**Methods:**

In this retrospective study, we analyzed data from 2,675 STEMI patients admitted to our hospital between January 2014 and December 2021. Patients were stratified into two groups based on their natural logarithmic ADR (Ln ADR): a high Ln ADR group (≥3.998) and a low Ln ADR group (<3.998). Univariate and multivariate Cox regression analyses were performed to assess the association between Ln ADR levels and clinical outcomes, including all-cause mortality and new-onset stroke.

**Results:**

Over a mean follow-up period of 1,013 days (interquartile range: 466–1,449 days), the incidence of major adverse cardiovascular events (MACE) was significantly higher in the low Ln ADR group compared to the high Ln ADR group (20.87% vs. 12.33%, *P* < 0.001). This disparity was particularly evident in all-cause mortality (6.58% vs. 1.00%, *P* < 0.001) and new-onset stroke (4.19% vs. 0.90%, *P* < 0.001). Multivariate analysis revealed that low Ln ADR was an independent predictor of all-cause mortality (HR = 2.46, 95% CI: 1.25–4.81, *P* = 0.009) and new-onset stroke (HR = 2.93, 95% CI: 1.35–6.35, *P* = 0.006).

**Conclusions:**

Reduced ADR levels were independently associated with increased long-term all-cause mortality and new-onset stroke in STEMI patients following hospital discharge. These findings suggest that ADR may serve as a valuable prognostic marker for risk stratification in this population.

## Introduction

Coronary artery disease (CAD) remains the predominant cause of global morbidity and mortality (1, 2). Although significant progress has been made in the management of ST-elevation myocardial infarction (STEMI)—including timely reperfusion via percutaneous coronary intervention (PCI), advancements in acute cardiovascular care, and the adoption of more effective pharmacotherapies (3, 4)—the mortality and complication rates among post-discharge patients persist at concerning levels, especially in high-risk populations (5). Consequently, there is an urgent need to identify more reliable indicators for early recognition of high-risk patients and to optimize post-discharge management strategies.

Previous studies have established that both albumin (6–8) and D-dimer (9–11) are valuable predictors of long-term outcomes in patients with CAD. In recent years, the albumin-to-D-dimer ratio (ADR), an emerging biomarker, has gained recognition as a significant prognostic indicator, particularly in the context of cardiovascular diseases (12, 13). For instance, Li et al. (14) demonstrated in a study of 3,707 patients with heart failure and multivessel disease undergoing elective PCI that a higher D-dimer-to-albumin ratio was independently associated with major adverse cardiovascular events (MACE) and all-cause mortality in diabetic patients with ischemic heart failure. This finding underscores the potential of ADR as a reliable prognostic tool for this high-risk population. Additionally, Yuan et al. (15) highlighted a significant relationship between serum albumin and D-dimer levels in patients with non-valvular atrial fibrillation (NVAF), revealing an inverse correlation between D-dimer and albumin levels. Despite these advancements, no studies to date have specifically explored the prognostic utility of ADR in patients with STEMI.

Therefore, this study aims to investigate the prognostic role of the ADR in predicting long-term outcomes in patients with STEMI and to compare its predictive efficacy with that of albumin and D-dimer individually.

## Materials and methods

### Study design and participants

This retrospective cohort study aimed to assess the prognostic value of admission ADR in hospital survivors of STEMI. The study analyzed medical records of 3,205 adult patients consecutively admitted to the cardiac care unit (CCU) of the First Affiliated Hospital of Wenzhou Medical University between January 2014 and December 2021, all of whom were diagnosed with STEMI. Exclusions comprised patients who died during hospitalization (*n* = 155), those diagnosed with venous thromboembolism (VTE) or pulmonary embolism (PE) (*n* = 141), individuals with nephrotic syndrome (*n* = 65), and patients with missing D-dimer or albumin data (*n* = 169). After exclusions, 2,675 patients were included in the final analysis. Treatment and management decisions were made at the discretion of the attending cardiologists. As a retrospective study involving statistical analysis of a specific population without the use of individual patient identifiers, the Ethics Committee in Clinical Research of the First Affiliated Hospital of Wenzhou Medical University granted a waiver for informed consent.

### Data collection and definitions

For each patient, demographic characteristics, cardiovascular risk factors, medical history, laboratory data collected during hospitalization, initial vital signs, treatments, and discharge diagnoses were extracted from the electronic medical database. Blood samples, including measurements of albumin, D-dimer, C-reactive protein (CRP), alanine transaminase, aspartate transaminase, brain natriuretic peptide, thyroid-stimulating hormone, and lipid profile, were collected after overnight fasting within the first 24 h of hospitalization. Additional biochemical measurements, such as complete blood count, high-sensitivity troponin I, and creatinine, were obtained immediately upon admission. Patients were stratified into two groups based on their natural logarithmic albumin-to-D-dimer ratio (Ln ADR): a high Ln ADR group (≥3.998) and a low Ln ADR group (<3.998). ST-elevation myocardial infarction (STEMI) was diagnosed according to the Third Universal Definition of Myocardial Infarction (16). GRACE scores (https://www.outcomes-umassmed.org/grace/acs_risk/) were calculated separately for all patients.

### Follow-up and study endpoint

Outcome data were collected from hospital records or through direct interactions and telephone follow-ups with patients or their next of kin. The mean follow-up duration was 1,013 days (interquartile range: 466–1,449 days). MACE were defined as a composite endpoint, including non-fatal acute coronary syndrome (ACS), unplanned coronary revascularization (UCR), hospitalization for heart failure (HFH), stroke, and all-cause mortality. Non-fatal ACS was defined as the clinical diagnosis of unstable angina, STEMI or non-STEMI. UCR was defined as unplanned PCI or surgical bypass after the index angiography or during the schedule of treatment. HFH was defined as an inpatient treatment with a primary or secondary diagnosis of heart failure identified using ICD-10 code I50.x. Stroke was defined as an acute neurological deficit attributed to ischemic or hemorrhagic causes, confirmed by computed tomography (CT) scan or magnetic resonance imaging (MRI).

### Statistical analysis

Statistical analyses were performed using chi-square tests and independent sample t-tests to compare baseline characteristics between the two groups. Kaplan–Meier survival curves were constructed to analyze patient survival rates, and log-rank tests were used to assess differences between the groups. Multivariate analysis was conducted using Cox proportional hazards regression models to evaluate the relationship between Ln ADR levels and outcomes, including all-cause mortality and new-onset stroke. Three models were employed: Model 1 was unadjusted, Model 2 adjusted for age and gender, and Model 3 further adjusted for potential confounders, such as atrial fibrillation, hypertension, diabetes, hyperlipidemia, smoking, alcohol consumption, prior stroke, primary PCI, Killip classification, admission creatinine levels, low-density lipoprotein cholesterol, brain natriuretic peptide, fibrinogen, alanine aminotransferase, CRP, hemoglobin, discharge medications, and left ventricular ejection fraction. Additionally, receiver operating characteristic (ROC) curve analysis was performed, and the area under the curve (AUC) was calculated to compare the predictive performance of Ln ADR, albumin, and D-dimer for all-cause mortality and new-onset stroke in patients with STEMI. ROC curve analysis for all-cause mortality of Ln ADR, GRACE score and Ln ADR combined with GRACE score were also performed.

## Results

### General clinical data

**Figure 1:**
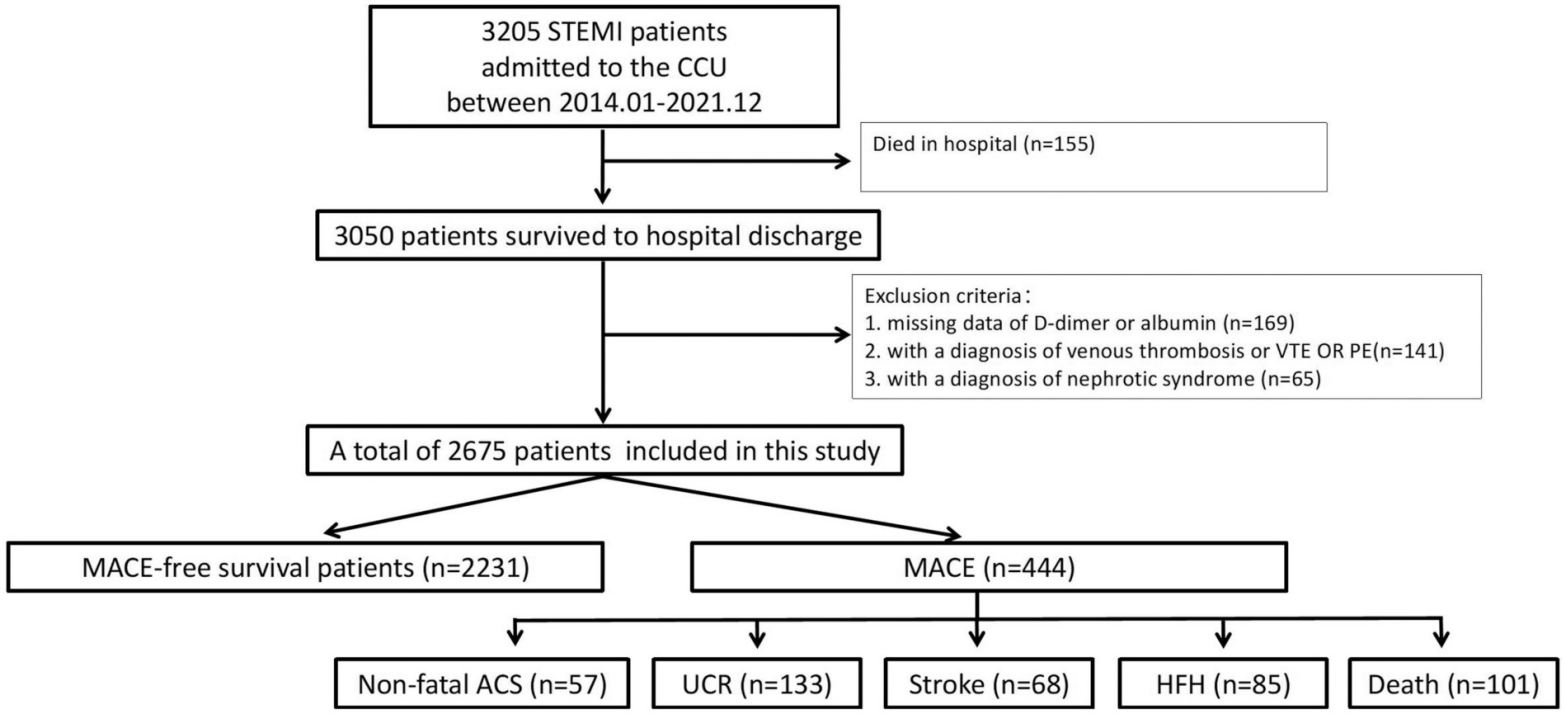
Study flowchart. STEMI, ST-elevation myocardial infarction; CCU, cardiac care unit; VTE, thromboembolism; PE, pulmonary embolism; MACE, adverse cardiovascular events; ACS, acute coronary syndrome; UCR, unplanned coronary revascularization; HFH, heart failure hospitalization.

As shown in Figure 1 during the study period, 3,205 STEMI patients were admitted to the CCU, of whom 3,050 (93.8%) survived to discharge. After excluding 375 patients who did not meet the inclusion criteria, the final study population comprised 2,675 participants (79.96% male), with a mean age of 63.13 ± 13.21 years. Baseline characteristics are presented in Table 1. Compared to the high Ln ADR group, the low Ln ADR group consisted of older patients (66.75 ± 12.80 vs. 59.52 ± 12.61, *P* < 0.001), a lower proportion of males (75.47% vs. 84.45%, *P* < 0.001), a higher GRACE score (152.63 ± 34.85 vs. 131.39 ± 27.81, *P* < 0.001), and lower proportions of current smokers (44.73% vs. 52.09%, *P* < 0.001) and drinkers (20.72% vs. 27.28%, *P* < 0.001). Additionally, the low Ln ADR group had higher proportions of atrial fibrillation (10.02% vs. 4.93%, *P* < 0.001) and prior stroke (8.23% vs. 4.19%, *P* < 0.001).

**Table 1:** Baseline characteristics of the study population stratified by the admission Ln ADR levels.

Variables	Total (*n* = 2,675)	High Ln ADR (≥3.998; *n* = 1,338)	Low Ln ADR (<3.998; *n* = 1,337)	*P*-value
Demographic				
Age (years)	63.13 ± 13.21	59.52 ± 12.61	66.75 ± 12.80	<0.001
Male	2,139 (79.96%)	1,130 (84.45%)	1,009 (75.47%)	<0.001
GRACE Score	142.01 ± 33.26	131.39 ± 27.81	152.63 ± 34.85	<0.001
Medical history				
Hypertension	1,531 (57.23%)	744 (55.61%)	787 (58.86%)	0.089
Diabetes mellitus	662 (24.75%)	333 (24.89%)	329 (24.61%)	0.866
Atrial fibrillation	200 (7.48%)	66 (4.93%)	134 (10.02%)	<0.001
Hyperlipidemia	1,085 (40.56%)	571 (42.68%)	514 (38.44%)	0.026
Current smoker	1,295 (48.41%)	697 (52.09%)	598 (44.73%)	<0.001
Current drinking	642 (24.00%)	365 (27.28%)	277 (20.72%)	<0.001
Prior MI	66 (2.47%)	27 (2.02%)	39 (2.92%)	0.134
Prior PCI	129 (4.82%)	55 (4.11%)	74 (5.53%)	0.086
Prior Stroke	166 (6.21%)	56 (4.19%)	110 (8.23%)	<0.001
Presentation characteristics				
PPCI	2,381 (89.01%)	1,268 (94.77%)	1,113 (83.25%)	<0.001
SBP (mmHg)	124.55 ± 21.38	125.34 ± 20.68	123.76 ± 22.04	0.055
DBP (mmHg)	76.08 ± 14.55	77.88 ± 14.65	74.29 ± 14.23	<0.001
Heart rate (beats/min)	81.84 ± 17.51	81.12 ± 15.85	82.56 ± 19.01	0.034
Killip class >1	561 (20.97%)	166 (12.41%)	395 (29.54%)	<0.001
Heparin use after PCI	125 (4.67%)	54 (4.04%)	71 (5.31%)	0.120
LVEF (%)	48.62 ± 8.75	49.92 ± 8.13	47.32 ± 9.15	<0.001
Laboratory parameters				
Albumin (g/L)	36.91 ± 3.79	38.07 ± 3.23	35.76 ± 3.94	<0.001
D-dimer (mg/L)	0.88 ± 0.74	0.377 ± 0.15	1.38 ± 0.75	<0.001
Fibrinogen (g/L)	3.63 ± 1.15	3.41 ± 0.90	3.85 ± 1.32	<0.001
ALT (U/L)	65.71 ± 89.54	59.95 ± 39.27	71.47 ± 120.11	<0.001
AST (U/L)	266.50 ± 216.86	270.40 ± 190.01	262.59 ± 240.77	0.352
Hb (g/L)	131.81 ± 18.38	136.49 ± 16.23	127.13 ± 19.19	<0.001
CRP (mg/L)	21.70 ± 27.84	16.25 ± 23.30	26.58 ± 30.55	<0.001
NLR	9.82 ± 6.53	9.33 ± 5.89	10.31 ± 7.09	<0.001
BNP (ng/L)	342.98 ± 743.38	197.58 ± 282.11	488.48 ± 999.99	<0.001
TSH (mIU/L)	1.22 ± 2.31	1.16 ± 2.68	1.29 ± 1.87	0.140
Creatinine (mmol/L)	81.45 ± 81.84	71.05 ± 58.69	91.86 ± 98.71	<0.001
LDL-c (mmol/L)	3.07 ± 1.01	3.11 ± 0.96	3.03 ± 1.05	0.027
Lactate (mmol/L)	2.63 ± 1.44	2.59 ± 1.20	2.67 ± 1.65	0.177
Medication use at discharge				
Aspirin	2,512 (93.91%)	1,286 (96.11%)	1,226 (91.70%)	<0.001
Clopidogrel or Ticagrelor	2,542 (95.03%)	1,307 (97.68%)	1,235 (92.37%)	<0.001
Anticoagulant	195 (7.29%)	80 (5.98%)	115 (8.60%)	0.009
Beta-blocker	2,116 (79.10%)	1,131 (84.53%)	985 (73.67%)	<0.001
ACEI or ARB or ARNI	1,694 (63.33%)	930 (69.51%)	764 (57.14%)	<0.001

Categorical variables are expressed as *n* (%); continuous variables as mean ± SD.

MI, myocardial infarction; PPCI, primary percutaneous coronary intervention; SBP, systolic blood pressure; DBP, diastolic blood pressure; LVEF, left ventricular ejection fraction; ALT, alanine transaminase; AST, aspartate aminotransferase; Hb, hemoglobin; CRP, C-reactive protein; NLR, Neutrophils lymphocyte ratio; BNP, brain natriuretic peptide; TSH, thyroid stimulating hormone; LDL-c, low-density lipoprotein cholesterol; ACEI, angiotensin-converting enzyme inhibitor; ARB, angiotensin receptor blocker, ARNI, angiotensin receptor neprilysin inhibitor.

The low Ln ADR group also had a lower proportion of patients receiving primary PCI (83.25% vs. 94.77%, *P* < 0.001), lower diastolic blood pressure (74.29 ± 14.23 vs. 77.88 ± 14.65 mmHg, *P* < 0.001), higher heart rates (82.56 ± 19.01 vs. 81.12 ± 15.85 beats/min, *P* = 0.034), higher Killip class (>1) (29.54% vs. 12.41%, *P* < 0.001), and lower left ventricular ejection fraction (47.32 ± 9.15 vs. 49.92 ± 8.13%, *P* < 0.001). In terms of laboratory parameters, the low Ln ADR group exhibited higher levels of D-dimer, fibrinogen, alanine transaminase, brain natriuretic peptide, neutrophil-to-lymphocyte ratio (NLR), CRP, and creatinine (*P* < 0.05 for all), while displaying lower levels of albumin, hemoglobin, and low-density lipoprotein cholesterol compared to the high Ln ADR group (*P* < 0.05 for all).

Regarding discharge medications, the use of aspirin, clopidogrel or ticagrelor, beta-blockers, and angiotensin-converting enzyme inhibitors or angiotensin receptor blockers or angiotensin receptor neprilysin inhibitors was more frequent in the low Ln ADR group, whereas anticoagulants were more frequently used in the high Ln ADR group (*P* < 0.05 for all). There were no significant differences between the two groups in terms of hypertension, diabetes mellitus, prior myocardial infarction (MI), prior PCI, systolic blood pressure, aspartate transaminase, thyroid-stimulating hormone, or lactate levels. Notably, CRP and NLR levels were significantly higher in the low Ln ADR group, suggesting a more pronounced inflammatory response in these patients.

### Clinical endpoint and follow-up

As shown in Table 2, the incidence of major adverse cardiovascular events (MACE) was significantly higher in the low Ln ADR group compared to the high Ln ADR group (20.87% vs. 12.33%, *P* < 0.001). Specifically, the incidence rates of new-onset stroke and all-cause mortality were markedly elevated in the low Ln ADR group (4.19% vs. 0.09%, *P* < 0.001; and 6.58% vs. 1.00%, *P* < 0.001, respectively). While the incidence rates of UCR was reduced in the low Ln ADR group (4.11% vs. 5.83%, *P* = 0.041). Further analysis revealed that the primary causes of new-onset stroke in this STEMI patient cohort included cerebral infarction due to atherosclerosis (*n* = 48, 70.59%), atrial fibrillation (*n* = 9, 13.23%), ventricular thrombosis (*n* = 1, 1.47%), cerebral hemorrhage (*n* = 7, 10.29%), and unknown causes (*n* = 3, 4.41%) (see Supplementary Material). These findings indicate that atherosclerosis is the predominant etiology of new-onset stroke in STEMI patients.

**Table 2:** Major adverse cardiovascular events between the two groups based on the admission Ln ADR levels.

Outcomes	Total (*n* = 2,675)	High Ln ADR (≥3.998; *n* = 1,338)	Low Ln ADR (<3.998; *n* = 1,337)	*P*-value
MACE	444 (16.60%)	165 (12.33%)	279 (20.87%)	<0.001
Non-fatal ACS	57 (2.13%)	24 (1.79%)	33 (2.47%)	0.227
UCR	133 (4.97%)	78 (5.83%)	55 (4.11%)	0.041
Stroke	68 (2.54%)	12 (0.90%)	56 (4.19%)	<0.001
HFH	85 (3.18%)	38 (2.84%)	47 (3.52%)	0.319
All-cause mortality	101 (3.78%)	13 (1.00%)	88 (6.58%)	<0.001

Categorical variables are expressed as *n* (%).

Ln ADR, natural logarithmic albumin-to-D-dimer ratio ratio; MACE, major adverse cardiovascular events; ACS, acute coronary syndromes; UCR, unplanned coronary revascularization; HFH, heart failure hospitalization.

### Association of Ln ADR levels with All-cause mortality and new-onset stroke

**Figure 2:**
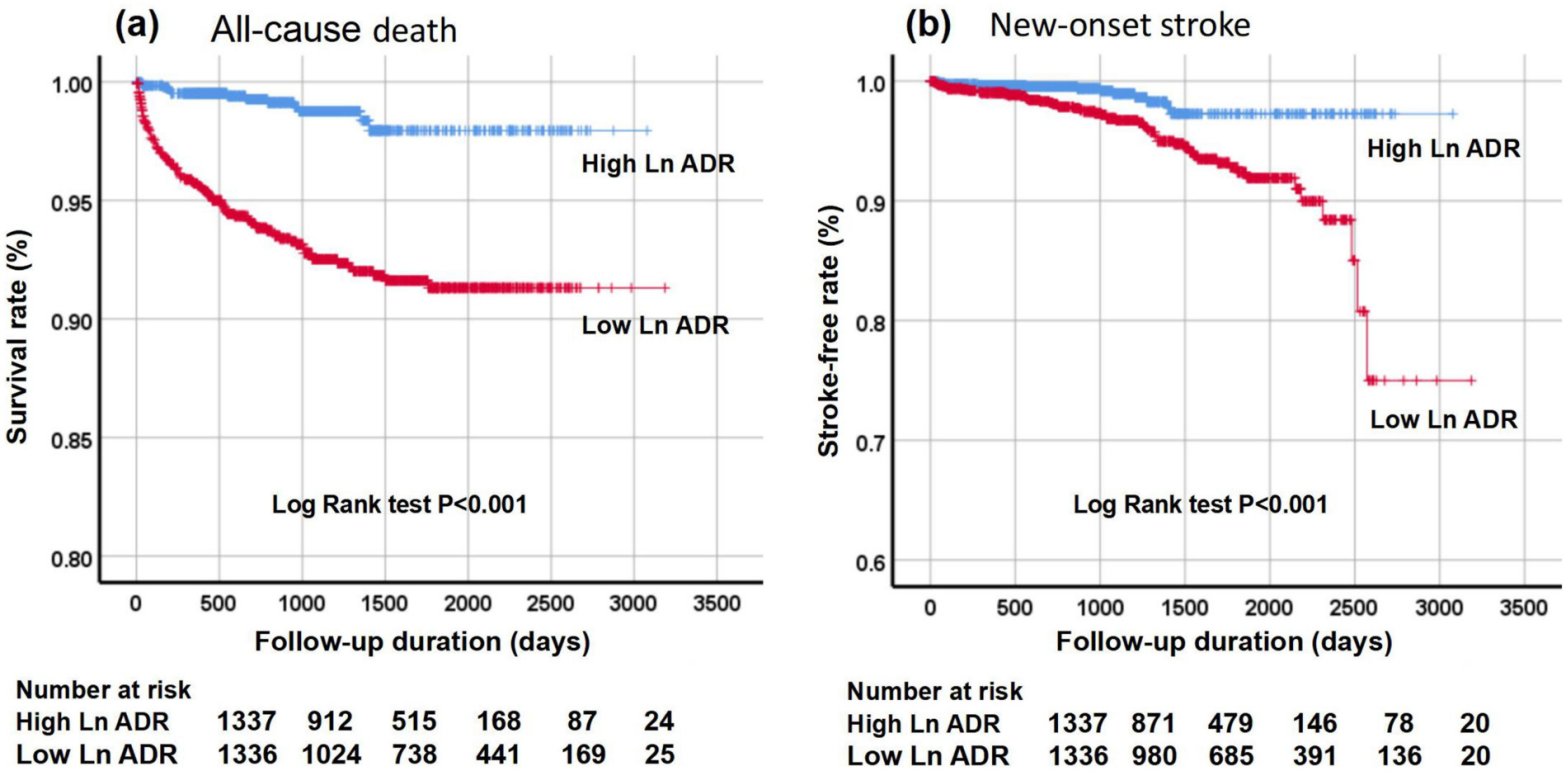
Kaplan–meier survival analysis for all-cause mortality **(a)** and new-onset stroke during follow-up **(b)** stratified by two Ln ADR groups. Kaplan–Meier analysis of all-cause mortality and new-onset stroke revealed poorer outcomes in the low Ln ADR group (red line) compared to the high Ln ADR group (blue line). Ln ADR, natural logarithmic albumin-to-D-dimer ratio ratio.

As illustrated in Figure 2, the cumulative incidence rates of all-cause mortality and new-onset stroke were significantly higher in the low Ln ADR group compared to the high Ln ADR group (*P* < 0.001). Multivariate Cox regression analysis (Table 3) revealed that in the unadjusted model (Model 1), the risks of all-cause mortality and new-onset stroke in the low Ln ADR group were 6.31 times (95% CI: 3.53–11.29, *P* < 0.001) and 3.33 times (95% CI: 1.78–6.24, *P* < 0.001) higher, respectively, than those in the high Ln ADR group. After adjusting for age and gender (Model 2), these risks decreased to 3.95 times (95% CI: 2.19–7.12, *P* < 0.001) and 2.98 times (95% CI: 1.57–5.65, *P* = 0.001), respectively. Further adjustments for potential confounders (Model 3) demonstrated that the risks of all-cause mortality and new-onset stroke in the low Ln ADR group remained significantly elevated, at 2.46 times (95% CI: 1.25–4.81, *P* = 0.009) and 2.93 times (95% CI: 1.35–6.35, *P* = 0.006), respectively. These findings indicate that Ln ADR level is an independent predictor of all-cause mortality and new-onset stroke in this patient population.

**Table 3:** Association of admission Ln ADR levels with all-cause mortality and new-onset stroke during follow-up.

	All-cause mortality			New-onset stroke		
	HR	95% CI	*P*-value	HR	95% CI	*P*-value
Model 1						
High Ln ADR	reference	reference		reference	reference	
Low Ln ADR	6.31	3.53–11.29	<0.001	3.33	1.78–6.24	<0.001
Model 2						
High Ln ADR	reference	reference		reference	reference	
Low Ln ADR	3.95	2.19–7.12	<0.001	2.98	1.57–5.65	0.001
Model 3						
High Ln ADR	reference	reference		reference	reference	
Low Ln ADR	2.46	1.25–4.81	0.009	2.93	1.35–6.35	0.006

Model 1 was unadjusted.

Model 2 was adjusted for age, sex.

Model 3 was adjusted for age, sex, atrial fibrillation, hypertension, diabetes, hyperlipidemia, current smoker, current drinking, prior stroke, PPCI, Killip class at admission, creatinine, LDL-c, BNP, fibrinogen, ALT, CRP, Hb, medical treatments at discharge (aspirin, clopidogrel or ticagrelor, anticoagulant, ACEI or ARB or ARNI, Beta-blocker) and LVEF.

Ln ADR, natural logarithmic albumin-to-D-dimer ratio ratio; PPCI, primary percutaneous coronary intervention; LDL-c, low-density lipoprotein cholesterol; BNP, brain natriuretic peptide; ALT, alanine transaminase; CRP, C-reactive protein; Hb, hemoglobin; ACEI, angiotensin-converting enzyme inhibitor; ARB, angiotensin receptor blocker; ARNI, angiotensin receptor neprilysin inhibitor; LVEF, left ventricular ejection fraction.

### Comparison of predictive efficacy of Ln ADR, albumin, and D-dimer

**Figure 3:**
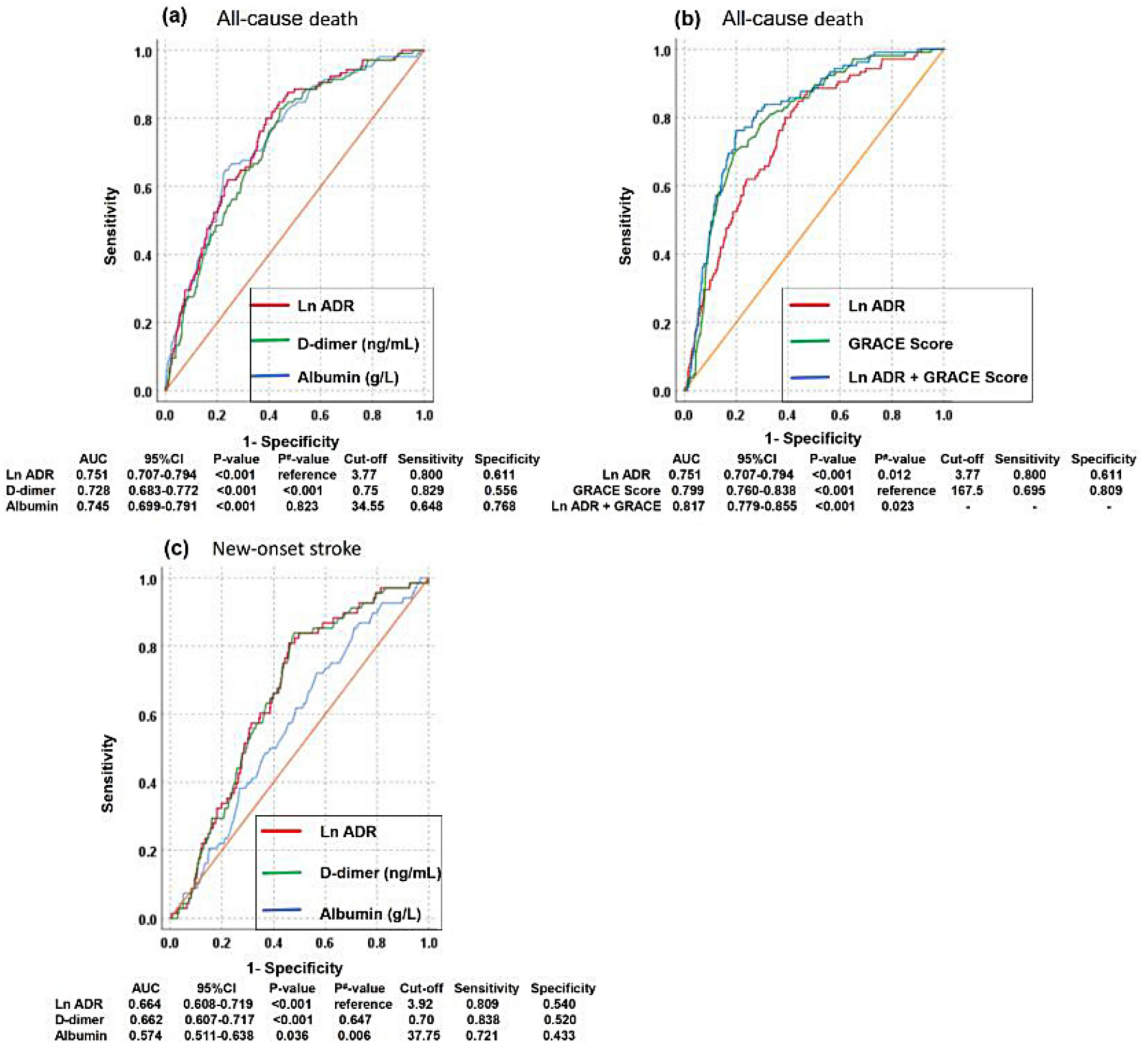
Receiver operating characteristic (ROC) curves of Ln ADR, albumin and D-dimer for all-cause mortality **(a)**, ROC of Ln ADR, GRACE score and Ln ADR + GRACE score for all-cause mortality **(b)** and ROC of Ln ADR, albumin and D-dimer for new-onset stroke **(c)** during follow-up. AUC, area under the curve; Ln ADR, natural logarithmic albumin-to-D-dimer ratio ratio.

The ROC curve for all-cause mortality (Figure 3a) demonstrated that the area under the curve (AUC) for Ln ADR was slightly higher than that for albumin (0.751 vs. 0.745, *P* = 0.823) and significantly higher than that for D-dimer (0.751 vs. 0.728, *P* < 0.001). A cut-off value of 3.77 for Ln ADR yielded a specificity of 0.611 and a sensitivity of 0.800 in predicting all-cause mortality in STEMI patients. Moreover, the AUCs (95% CIs) (Figure 3b) for Ln ADR combined with GRACE score and GRACE score were 0.817 (0.779, 0.855) and 0.799 (0.760, 0.838), respectively (*p* = 0.023). Analysis showed that the predictive ability for all-cause mortality of Ln ADR combined with GRACE score was better than that of GRACE score alone. The ROC curve for new-onset stroke (Figure 3c) revealed that the AUC for Ln ADR was slightly higher than that for D-dimer (0.664 vs. 0.662, *P* = 0.647) and significantly higher than that for albumin (0.664 vs. 0.574, *P* = 0.006). A cut-off value of 3.92 for Ln ADR provided a specificity of 0.540 and a sensitivity of 0.809 in predicting new-onset stroke in STEMI patients.

In conclusion, Ln ADR demonstrates superior predictive efficacy compared to albumin or D-dimer alone for both all-cause mortality and new-onset stroke in STEMI patients. These findings suggest that Ln ADR serves as a more effective prognostic indicator, aiding clinicians in enhanced risk stratification and management.

## Discussion

### Main findings

The key findings of this study are as follows: (1) Patients with low Ln ADR levels exhibited a significantly higher incidence of MACE compared to those with high Ln ADR levels; (2) all-cause mortality and new-onset stroke were the primary contributors to the observed disparity in MACE incidence between the two groups; and (3) decreased ADR levels were identified as independent predictors of long-term all-cause mortality and new-onset stroke in STEMI patients following hospital discharge. The ROC curve revealed that Ln ADR had a better ability than D-dimer to predict all-cause mortality and had a better ability than albumin to predict new-onset stroke in hospital-discharged STEMI patients, and its use in combination with GRACE score could improve the predictive ability for all-cause mortality of GRACE score alone. (4) The incidence rate of UCR was reduced in the low Ln ADR group.

### Albumin and D-dimer influence the prognosis in CAD patients

Albumin, the most abundant plasma protein in the human body, serves as a vital nutritional marker, the primary determinant of colloid osmotic pressure, and a key regulator of fluid balance (17). It fulfills several critical physiological roles, including maintaining vascular integrity, regulating cholesterol transport, facilitating anticoagulation, and providing antioxidative effects. Moreover, albumin has been increasingly recognized as a significant biomarker in the identification and management of cardiovascular diseases (18). Studies have shown that low albumin levels are strongly associated with an elevated risk of major adverse cardiac events, such as all-cause mortality, nonfatal myocardial infarction, and nonfatal stroke, in patients with CAD (10–12).

D-dimer, a degradation product of fibrin, serves as a marker of active coagulation and fibrinolysis, playing a crucial role in diagnosing and monitoring conditions such as deep vein thrombosis, pulmonary embolism, and disseminated intravascular coagulation (19). Recent evidence suggests that elevated baseline D-dimer levels significantly increase the risk of all-cause mortality in CAD patients and are associated with poor long-term outcomes (13–15).

The ADR, which integrates both biomarkers, has emerged as a promising prognostic indicator in recent years (12, 13), particularly in the context of cardiovascular diseases (14, 15). Our findings are consistent with previous research, highlighting the strong association of albumin and D-dimer levels with cardiovascular disease outcomes. In this study, patients with low Ln ADR exhibited a higher-risk and more fragile clinical phenotype, characterized by worse hemodynamic status, higher inflammatory burden, and lower rates of primary PCI. Therefore, ADR may partially reflect overall disease severity or frailty rather than representing a completely independent biological pathway. However, this study is the first to propose ADR as a comprehensive predictive tool with enhanced efficacy for all-cause mortality and new-onset stroke in STEMI patients. Importantly, we demonstrate that ADR outperforms either albumin or D-dimer alone in predicting these critical clinical endpoints, offering clinicians a simple yet effective tool to improve risk stratification and prognosis assessment in STEMI patients.

### Coronary artery disease and stroke

Stroke is the second leading cause of death globally. There is a well-established association between CAD and stroke, as both conditions share numerous common risk factors and pathological mechanisms (20–23). One study reported that the prevalence of asymptomatic CAD among stroke patients is relatively high, ranging from 20% to 40%, while the short-term incidence of cardiac events in these patients is between 2% and 5%. Moreover, the long-term risk of cardiac events in stroke patients is at least twice that of age-matched controls (20). Another study found that patients with acute ischemic stroke (AIS), particularly those with large-artery atherosclerosis, are more likely to have concurrent obstructive CAD (21). However, most existing research has focused on the risk of newly developed CAD in stroke patients, with limited studies exploring the risk of new-onset stroke in CAD patients. Identifying predictive indicators for stroke in CAD patients could significantly improve clinical strategies for stroke prevention in this population.

A study highlighted that arterial occlusion is the primary cause of most ischemic strokes (24), which aligns with our findings that identified atherosclerosis as the leading cause of new-onset stroke in STEMI patients (*n* = 48, 70.59%). This suggests a consistent relationship between arterial blockages and stroke occurrences in both ischemic stroke and STEMI populations. Furthermore, previous studies have established a strong link between atherosclerosis and inflammation (25, 26). In our study, we observed significantly higher levels of CRP and NLR in the low Ln ADR group. CRP, an acute-phase reactant protein and marker of inflammation, is often elevated in systemic inflammatory conditions. A prospective study demonstrated that CRP is a significant predictor of first-ever ischemic stroke (27). NLR, a novel inflammatory marker, has recently gained attention for its ability to predict cardiovascular events. Research indicates that elevated NLR levels are associated with increased instability and inflammatory activity of atherosclerotic plaques (28–30).

The elevated levels of these inflammatory markers in the low Ln ADR group align with our statistical findings, which show a higher probability of new-onset stroke following STEMI in this group. Specifically, patients in the low Ln ADR group exhibited higher CRP and NLR levels, indicating a more pronounced inflammatory response. This heightened inflammatory state may accelerate the instability of atherosclerotic plaques, increasing the risk of plaque rupture and thrombosis, which could contribute to the higher incidence of new-onset stroke post-STEMI. Additionally, low serum albumin levels are often associated with systemic inflammation (31), further corroborating our findings.

### Reduced UCR incidence rate in the low Ln ADR group

This study identified a seemingly paradoxical association between a lower Ln ADR and higher all-cause mortality coupled with a lower rate of UCR. We propose that this finding is not contradictory but can be rationally explained by the interplay of competing risks, distinct clinical phenotypes, and differences in patient behavior and adherence.

The most compelling explanation for this observation lies in the concept of competing risks. Patients in the low Ln ADR group exhibited significantly higher mortality, primarily from non-ischemic causes such as fatal stroke, progressive heart failure, or malignant arrhythmias. These competing fatal events likely precluded the occurrence of subsequent UCR, as these patients died before their coronary disease could progress to a point necessitating repeat revascularization. This phenomenon, where a higher risk of one event (death) reduces the observed incidence of another (UCR), is well-established in methodological literature and is optimally analyzed using models that account for competing risks, such as the Fine and Gray subdistribution hazard model (32, 33).

Furthermore, the Ln ADR biomarker effectively stratifies patients into distinct clinical phenotypes with differing underlying risk profiles and consequent treatment approaches. The high Ln ADR group represents a more robust phenotype with better nutritional and inflammatory status. Their superior long-term survival permits the manifestation of later-stage coronary-specific complications, such as in-stent restenosis or progression of native vessel disease, which commonly present as ischemic events requiring UCR (34, 35). Conversely, the low Ln ADR group is characterized by a frail, high-risk phenotype with signs of chronic inflammation, hypercoagulability, and worse cardiac function. For these patients, clinical management is often more conservative. When facing recurrent symptoms, the high perceived risk of procedural complications (e.g., contrast-induced nephropathy, worsening heart failure) may lead clinicians to opt for intensified medical therapy rather than invasive intervention (36). Moreover, their clinical course is dominated by acute, often fatal, non-ischemic events (e.g., stroke, pump failure) that result in death or hospitalization but are not typically amenable to revascularization (35).

An additional layer of explanation involves risk perception and adherence patterns, particularly noted in the younger, high Ln ADR cohort. Although younger age is generally associated with better outcomes, it can also correlate with underestimation of long-term risk. This group reported higher rates of smoking and alcohol use at baseline, and long-term adherence to both lifestyle modifications and secondary prevention pharmacotherapy (e.g., statins, antiplatelet agents) may be suboptimal in younger patients due to a perceived state of wellness, concerns about side effects, or psychosocial factors (37, 38). This persistent exposure to risk factors, potentially compounded by less intensive long-term lipid management (as suggested by a trend towards higher LDL-c levels), can drive the progression of atherosclerosis and in-stent restenosis, thereby increasing the long-term risk of ischemic events requiring revascularization.

In summary, the inverse relationship between mortality and UCR in the low Ln ADR group is not indicative of a protective effect but rather reflects a shift in the mode of adverse events. This group is susceptible to early, fatal non-ischemic complications, which compete with and truncate the natural history of coronary artery disease, thereby artifactually reducing the observed rate of UCR. However, it should be emphasized that this study did not employ a formal competing risk model for analysis, and the findings are of hypothesis-generating significance only.

### Study limitations

Although our study highlights the significant predictive advantage of the ADR for all-cause mortality and new-onset stroke in STEMI patients, several limitations must be acknowledged and addressed in future research. First, this study is a retrospective, single-center investigation, therefore, a larger sample size with cross-center comparisons is required to validate the results further. And in this study, we excluded patients who died during hospitalization, those diagnosed with venous thromboembolism or pulmonary embolism, individuals with nephrotic syndrome, and patients with missing D-dimer or albumin data. This means that we will exclude the patients with the poorest prognosis. At the same time, we have excluded diseases that affect the levels of albumin and D2-polymer, as these diseases often also lead to poor prognosis. Therefore, the research results obtained from this potential selection bias may not be applicable to other populations. Second, while we adjusted for multiple confounders in the statistical analysis, certain factors—such as lifestyle variables (e.g., diet and exercise), socioeconomic status, and psychological state—were not accounted for, despite their known influence on CAD prognosis. Future studies should aim to collect and incorporate these variables to provide a more comprehensive assessment of the impact of ADR on CAD outcomes. Additionally, this study did not explore the underlying mechanisms by which ADR predicts CAD prognosis. Further research should include basic experimental studies to elucidate the mechanistic links between ADR and CAD prognosis, thereby strengthening the evidence base for its clinical application.

## Conclusions

In conclusion, this study demonstrates that the incidence of MACE in hospital-discharged STEMI patients is significantly higher in the low Ln ADR group compared to the high Ln ADR group, particularly in terms of all-cause mortality and new-onset stroke. Low ADR levels were identified as an independent predictor of long-term all-cause mortality and new-onset stroke in STEMI patients following hospital discharge. However, due to some limitations of this study, further research is necessary before ADR can be fully adopted as a key clinical indicator for assessing the prognosis of STEMI, enabling the identification of high-risk patients and guiding the optimization of management strategies.

## Data Availability

The raw data supporting the conclusions of this article will be made available by the authors, without undue reservation.
